# Reproductive phenology and pre-dispersal fruit predation in *Atriplex halimus* L. (*Chenopodiaceae*)

**DOI:** 10.1186/1999-3110-54-4

**Published:** 2013-08-12

**Authors:** Prado Romera, Francisca Fernández-Illescas, F Javier J Nieva, Pilar Rodríguez-Rubio, Enrique Sánchez-Gullón, Adolfo F Muñoz-Rodríguez

**Affiliations:** 1 Departamento de Biología Ambiental y Salud Pública, Facultad de Ciencias Experimentales, Campus de El Carmen, 21071 Huelva, Spain; 2 Departamento de Química y Ciencia de los Materiales, Facultad de Ciencias Experimentales, Universidad de Huelva, Huelva, Spain; 3 Paraje Natural Marismas del Odiel, Agencia de Medio Ambiente, Junta de Andalucía, Huelva, Spain

**Keywords:** *Atriplex halimus*, Flowers, Fruit predation, Insects, Phenology

## Abstract

**Background:**

The flowering phenology pattern of Atriplex halimus was studied in a Mediterranean habitat in order to analyze protandry effectiveness. Fruit set evolution was recorded over two years and the impact of pre-dispersal predation by insects was also evaluated.

**Results:**

The flowering phenology coincided in 2006 and 2007, starting in mid-July and reaching full flowering at the end of August in both years. Inflorescences are composed of glomerules with 8.78 ± 2.79 male flowers and 4.57 ± 2.58 female flowers, with no significant differences in position on the inflorescence. The peaks of male and female flower anthesis were reached in mid-August, but the male maximum occurred one week before the female. Plants at the start of flowering only bear male flowers, but female flowers soon appear. Fruit set starts at the end of August; all the flowers were transformed into fruit by mid-September and their development continued to the beginning of October, when fruit structures had matured and began to drop. Fruit predation started at the end of September and reached maximum intensity in mid-October.

**Conclusions:**

At population level, male and female flowers seemed to open in the same weeks, but at plant and glomerule level male flowers opened one week before the females. Fruit predation levels were 62.42 and 43.14% in 2006 and 2007 respectively, with no significant differences between different parts of the inflorescence. And larvae of *Coleophoridae* were the most abundant predators.

**Electronic supplementary material:**

The online version of this article (doi:10.1186/1999-3110-54-4) contains supplementary material, which is available to authorized users.

## Background

*Atriplex* shrub species, known as saltbushes, inhabit arid regions and are frequently found in saline soils (McArthur and Sanderson 1984; Cañadas et al. 2010). *Atriplex halimus* L. is a Mediterranean and Macronesian scrub that grows in dry saline soils, and is a characteristic species of halo-nitrophilous vegetation (*Pegano-Salsoletea*). It is planted extensively in these areas for ornamental purposes and as a fodder shrub due to its tolerance of drought and salinity; it plays an important role in the rehabilitation of degraded zones (Le Houerou 1992; Kinet et al. 1998; Le Houerou 2000; Papanastasis et al. 2008) and has improved the quality of pastures in arid zones (Osman et al. 2006).

*Atriplex halimus* is a monoecious scrub that produces staminate flowers, with a whorl of 5 green sepals and 5 stamens, and female flowers with a single carpel enclosed within two opposite bracts which develop into dry fruit (achenes) surrounded by 2 valves, giving rise to a fruit structure that disperses as a unit. (Talamali et al. 2001,2003) studied floral polymorphism in the flowers of this species, observing other types sporadically including hermaphrodite flowers. Male and female flowers are set in glomerules formed by the concrescence of several dichasial cymes deployed along the axis of the branched inflorescence. Female flowers mainly develop in the proximal part and male flowers in the distal part of each glomerule (Talamali et al. 2001), and the sex ratio is affected by day length and light intensity (Talamali et al. 2003).

As a rule, in the *Chenopodiaceae* family plants are predominantly wind-pollinated (Blackwell and Powell 1981) and there is no evidence of self-incompatibility (Heyligers 2001). Some studies have focused on the phenology of this species in different areas (Talamali et al. 2003), and one describes protandry (Abbad and Benchaabane 2004) which is consistent with the high levels of outcrossing found in this species (Haddioui and Baaziz 2001; Ferchichi et al. 2006).

In this study we examine the flowering phenology pattern of *A. halimus* in a Mediterranean habitat, determining male and female flower development on three levels (population, plant and glomerule) in order to analyze protandry effectiveness.

The rates of fruit and seed predation are important parameters of the population dynamic of plants. However, there is a lack of information on fructification and seed predation despite their importance as factors for the population dynamic of *A. halimus*. In this study fruit set evolution was recorded over two years and the impact of pre-dispersal predation by insects was also evaluated.

## Materials and methods

**Figure 1:**
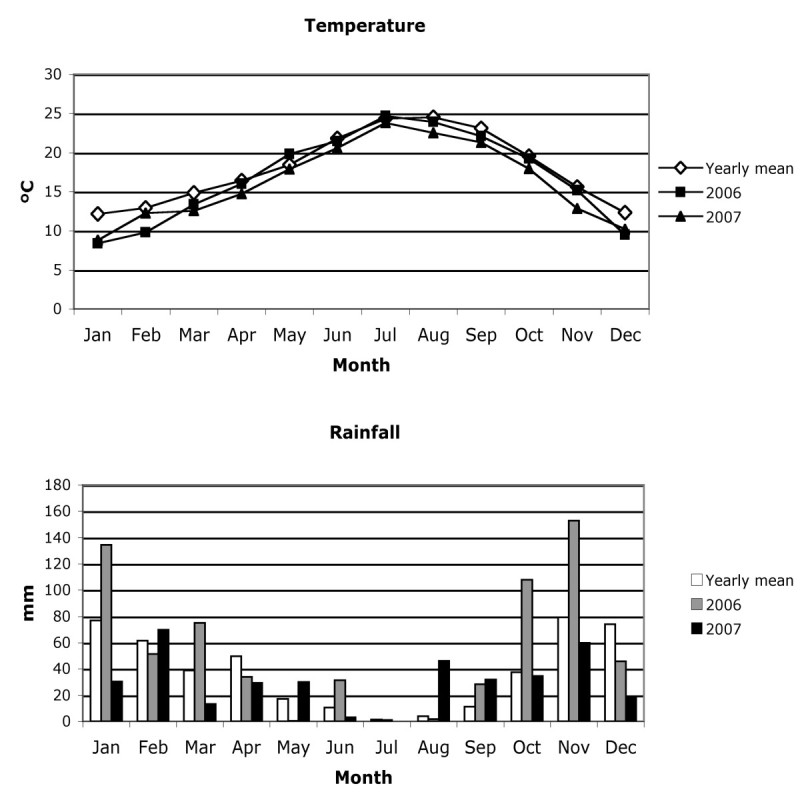
**Mean values of monthly temperature and precipitation for Moguer, and average monthly temperature and rainfall in 2006 and 2007.** Data recorded at the Moguer Meteorological Station.

*Atriplex halimus* plants were studied in a single population at the “Marismas del Odiel” Natural Park in Huelva (SW Spain). This population grows on a saline soil island surrounded by a tidal marsh in a virtually monospecific shrub community (37°13’53”N 6°57’50”W). See Fernández-Illescas et al. (2010) for cover values. This location has a typical Mediterranean climate with an average annual temperature of 18.0°C and an average annual rainfall of 460.9 mm, calculated from data recorded at the Moguer Meteorological Station, 12 km from the location (Rivas-Martínez and Rivas-Sáenz 1996–2009) (Figure 1). The study was carried out during 2006 and 2007, in which the annual temperatures were 17.0 and 16.3°C, and annual rainfall was 663.2 and 366.8 mm, respectively (Figure 1).

For population phenology, ten plants randomly distributed among the *Atriplex* population were marked at the beginning of 2006 and evaluated weekly to determine flowering stage on a scale of zero to ten that measured the proportion of inflorescences with open flowers. Ten inflorescences from different plants were picked randomly each week for laboratory analysis with loupe or microscope magnification. Three glomerules were randomly selected from each inflorescence, one from the upper, middle and lower parts, and analyzed independently to estimate the number of male and female flowers, and the evolution of fructification and predation.

Male flower phenology was studied in 2007 by counting the number of male flowers in each glomerule which marked three different developmental stages of the flower: B) male flower bud differentiated, A) male flower at anthesis liberating pollen, and S) senescent male flowers. As male flower bud differentiation occurs sequentially, and the male flowers fall from the glomerule and disappear after flower senescence, we estimated the average number of male flowers per glomerule by calculating the arithmetic mean for the two weeks when male flowering peaked (7 and 14 August 2007). The average values for different levels of inflorescence were also calculated.

Female flower phenology and fruit evolution were studied in 2006 and 2007 by counting the number of female flowers in each glomerule, which yielded three different developmental stages of the flower: B) female flower bud differentiated, A) female flower at anthesis protruding stigmas from the flower bracts, and F) fruiting female flower, showing prominent bract development. No senescent female flower was recorded as all the flowers observed had initiated fruit development. Once all female flowers are differentiated, and there is no flower drop, we assume that their numbers will remain stable until the onset of flower drop, with possible record variations due to sampling. In order to establish an approximate level of female flowers per glomerule, we considered the week in both years when flowering peaked (28 August 2006 and 18 September 2007) together with the week before and after. The global mean values for different levels of the inflorescence were calculated.

Each week the number of male flower per glomerule at each stage was calculated with the percentage of plants and glomerules with only male flowers opened, with only female flowers opened, with both types opened, and with no flower opened.

From 7 August (when the first female bloom peak occurs in both years) to 9 October (when the size of bracts and achenes was stable), 20 female flowers were randomly selected, two from each inflorescence, and bract length and width and ovule or achene length were measured. Their stigmas were removed and mounted on cotton blue lactophenol for microscopic examination, based on the observation of pollen tubes growing on each.

Predated fruits were also counted and the predators found on inflorescences were fixed for taxonomic identification by D. M. Huertas Dionisio, an insect specialist in the field (Huertas Dionisio 2007). The percentage of fruit predation was calculated weekly. To compare the figures obtained in both years, and on different parts of the inflorescence, only values above 0% were used in analysis.

Data normality was checked by using the Kolmogorov-Smirnov test. Statistical analysis was done via the ANOVA one-way analysis of variance (when data were normally distributed) or by a non-parametric test (Kruskal-Wallis) if not.

## Results

**Figure 2:**
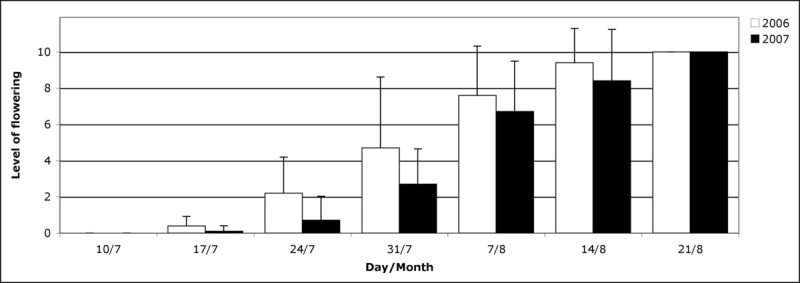
**Weekly mean and standard deviation values of flowering at population level (scale 0 to 10).**

The flowering phenology pattern in the *A. halimus* population in 2006 and 2007 was similar (Figure 2) with the start and full flowering dates virtually coinciding, 17 July and 21 August respectively; 2006 weekly values were higher, which could mean flowering started a little earlier than in 2007.

**Figure 3:**
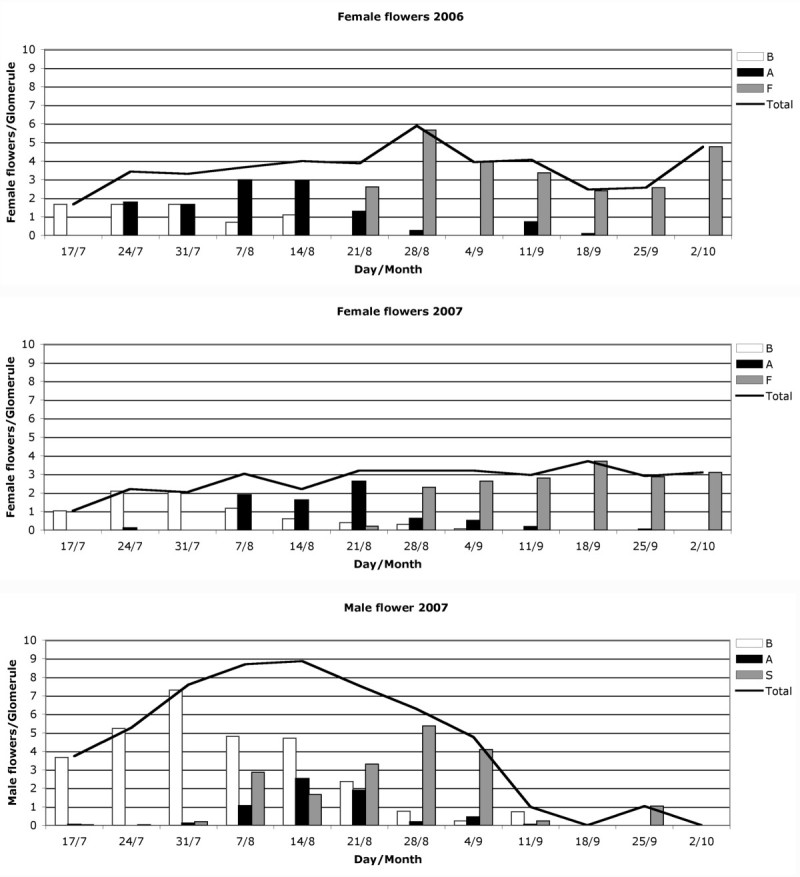
**Weekly values of flower number per glomerule.** Upper and middle graphs show the evolution of the female flowers during 2006 and 2007 respectively, separated according to their phases: B) bud, A) anthesis, F) fruit, and Total) number of female flowers. The lower graph shows the evolution of the male flowers in 2007 separated according to their phases: B) bud, A) anthesis, S) senescent, and Total) number of male flowers.

The weekly evolution of male and female flowers per glomerules and their ontogenic phases are shown separately (Figure 3). Male flower buds were differentiated in mid-July 2007; the first flowers opened in early August and by 28 August most of the male flowers were senescent. All had fallen from the glomerule by 1 October. Male flowers reached the peak of anthesis on 14 and 21 August that year, with a global mean of 8.78 ± 2.79 flowers per glomerules. By position on the inflorescence, the means were 9.95 ± 4.45, 8.50 ± 2.69 and 7.90 ± 2.43 for upper, medium and lower parts respectively, with no significant differences between these samples (Kruskal-Wallis: H = 1.25, p = 0.54). Female flowers buds also started differentiation in mid-July in 2006 and 2007, but the stigmas begin to protrude from the bracts earlier in 2006 (24 July) than in 2007 (7 August). The maximum number of female flowers in anthesis was reached on 7 and 14 August in 2006 and 21 August in 2007. Growth of the bracts, evidence of fruit development, started on 21 August in both years, and all the flowers had transformed into fruit structures by 18 September. No senescent female flowering was observed, so it was considered that 100% of flowers had developed into fruit.

**Figure 4:**
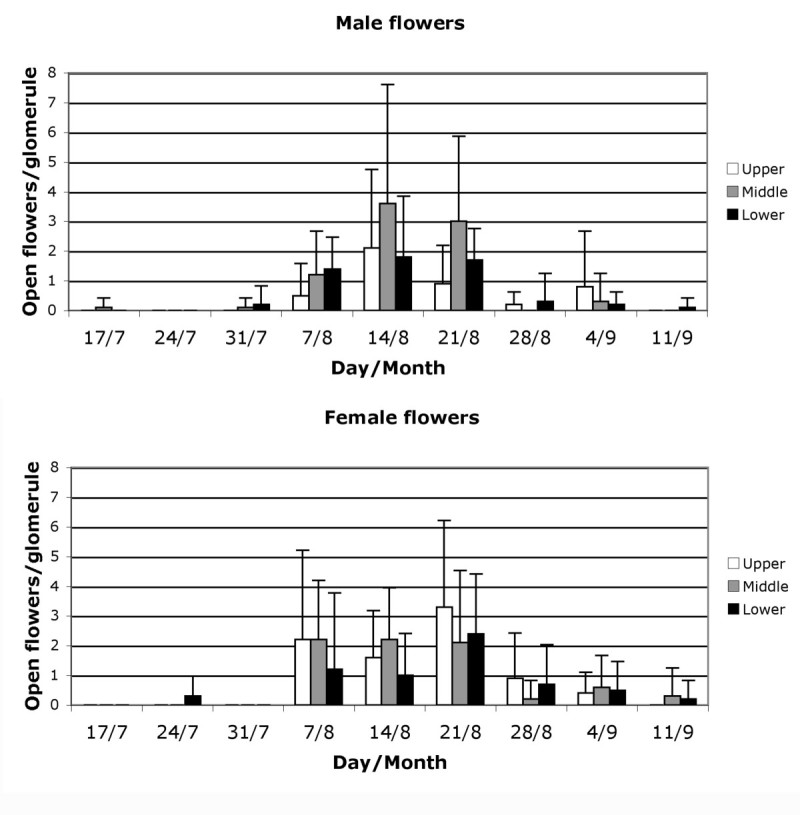
**Weekly mean values of flowers at anthesis per glomerule for three parts of the inflorescence observed in 2007: upper, middle and lower.**

The male and female anthesis period of the flowers (stage A) separated by position on the inflorescence was also studied (Figure 4). Male flowers started opening in the same week (7 August) in the three positions, and reached peak values in the same week (14 August). Most female flowers exposed their stigmas between 7 and 21 August in the three positions, but those of the upper and lower parts of the inflorescence reached the maximum on 21 August.

**Figure 5:**
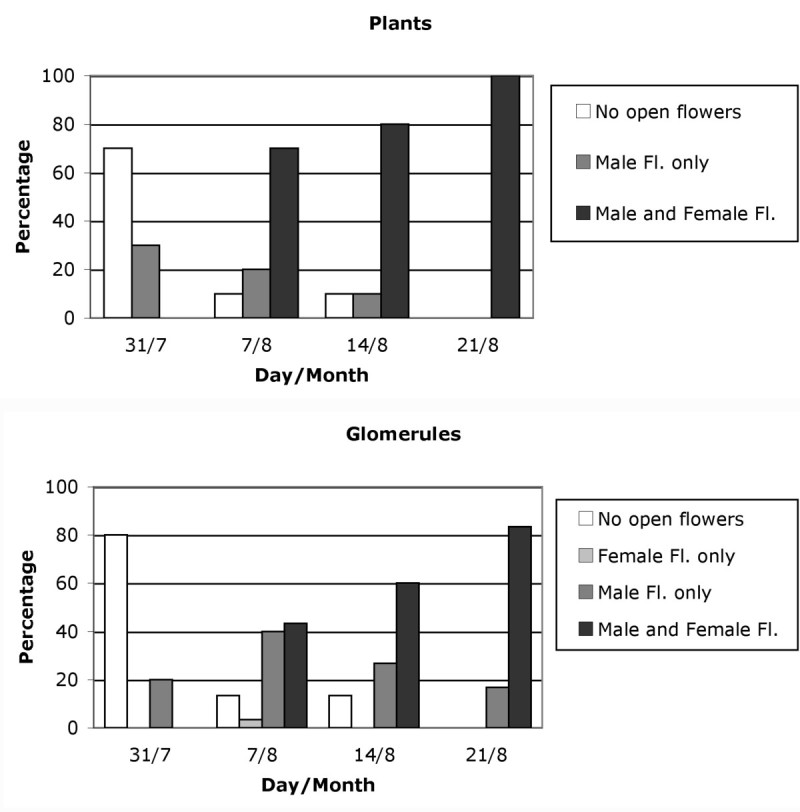
**Weekly percentage of plants and glomerules with: only male flowers opened (stages A and S); only female flowers opened (stages A and F); with flowers of both sexes opened; and with no flower opened.**

The plants at the start of flowering only had male flowers (30% of individuals on 31 July); one week later, 70% had male and female flowers (7 August); one week after they had reached 80% (14 August) and on 21 August 100% of plants had flowers of both sexes (Figure 5). No plant was recorded bearing only female flowers opened. There was always a proportion of glomerules with only male flowers opened, which recorded its maximum on 7 August (30%). By contrast, some glomerules examined on 7 August had only female flowers opened (3%).

**Figure 6:**
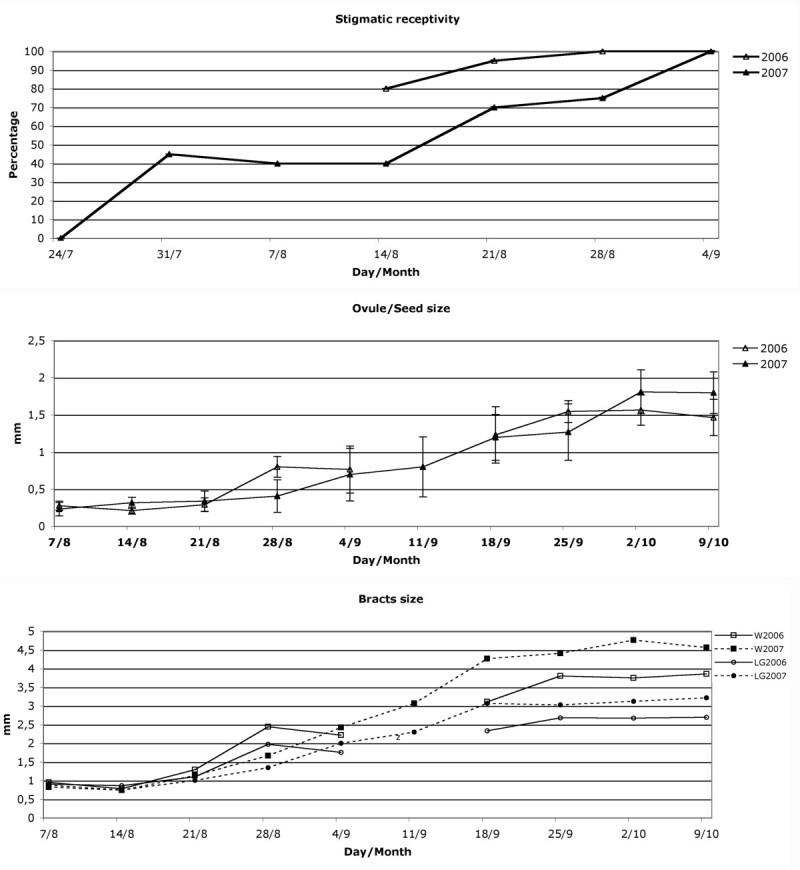
**Weekly stigmatic receptivity and ovule and bract development during the female flowering phase in 2006 and 2007.**

The changes in female flowers during this flowering period, from the beginning of protrusion of stigmas from bracts to 9 October 2007 (when fruit structures were mature and began to drop from glomerules) were also registered (Figure 6). On 31 July almost half of female flowers had pollen tubes on their stigmas. By weekly calculation, the percentage of stigmas with pollen tube growth reached 100% on 28 August 2006, and 4 September 2007, when most of the female flowers had passed the anthesis phase.

During anthesis the bract size was 0.91 ± 0.17 mm long by 0.95 ± 0.26 mm wide in 2006, and 0.75 ± 0.33 mm by 0.67 ± 0.19 mm in 2007, showing no significant differences in length (one-way ANOVA: F = 3.41, p = 0.73). Bracts in 2006 were significantly broader than in 2007 (one-way ANOVA: F = 14.66, p < 0.001). In this period, the size of the ovary was 0.27 ± 0.06 mm (2006) and 0.25 ± 0.07 mm (2007), with no significant difference between years (one-way ANOVA: F = 2.33, p = 0.13). In 2006, bracts began to grow on 21 August and reached maximum size on 25 September. Final bract size on 9 October was 2.70 ± 0.37 mm by 3.86 ± 0.65 mm, and 3.22 ± 0.58 mm by 4.57 ± 0.79 mm in 2007, significantly longer (one-way ANOVA: F = 11.16, p = 0.002) and broader (one-way ANOVA: F = 9.24, p = 0.004) than the previous year. On this date, achene size was 1.47 ± 0.25 mm in 2006 and 1.80 ± 0.28 mm in 2007, a significant difference between both years (one-way ANOVA: F = 15.40, p < 0.001).

**Figure 7:**
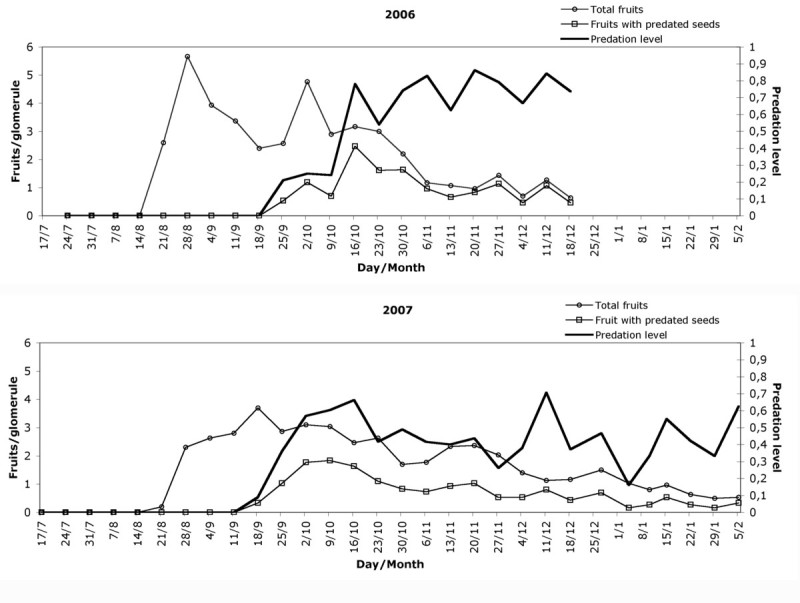
**Weekly total and predated fruits per glomerule and predation level.**

Maximum values of fruit per glomerule were recorded on 28 August 2006 and 18 September 2007 (Figure 7). The global mean number of fruits per glomerule on these dates, including the weeks before and after the peak, was 4.57 ± 2.58 (2006) and 3.19 ± 1.32 (2007), the differences being significant (Kruskal-Wallis: H = 18.51, p < 0.001). The glomerules from the upper, middle and lower levels in 2006 contained 4.07 ± 1.89, 4.87 ± 3.26 and 4.77 ± 2.42 fruits respectively, with no significant differences between the three (Kruskal-Wallis: H = 0.97, p = 0.61). In 2007 these values were 3.40 ± 1.43, 3.10 ± 1.09 and 3.07 ± 1.44 fruits, once again with no significant differences between them (Kruskal-Wallis H = 1.08; p = 0.58). From these peaks the number of fruits per glomerule decreased as a consequence of fruit structure separation from the glomerule. This liberation was faster in 2006 than in 2007.

Fruit predation was first observed on 25 September 2006 and on 18 September 2007 (Figure 7), reaching a maximum on 16 October in both years, with 78% (2006) and 66% (2007) of achenes predated. After this date, the level of predation varied weekly, making interpretation difficult. The probable cause was the process of fruit liberation and the intense bird predation observed in the field. The average of predated fruit by insects was 62.42% (2006) and 43.14% (2007), a significant difference (one-way ANOVA: F = 8.1, p = 0.008). There were no significant differences in predation levels between upper, middle and lower parts either in 2006 (one-way ANOVA: F = 0.54, p = 0.58) or in 2007 (one-way ANOVA: F = 1.46, p = 0.25). Fruiting and predation showed no significant correlation between them.

During the study (in September) some insect eggs were found near the ovaries of female flowers in *A. halimus*. Many *Coleophoridae* (*Lepidoptera*) larvae were located in the inflorescences in the last week of September and first days of October in both years. Some *Gelechiidae* (*Lepidoptera*) larvae were also found in September.

## Discussion

*A. halimus* is mainly considered a monoecious species which is occasionally dioecious. However, some authors have found individuals that present unisexual and hermaphrodite flowers so this species could be polygamous or, more precisely, trimonoecious (Talamali et al. 2001,2003). Our study observed no other flower type than the typical male and female flowers, although one female flower was found to contain abortive stamen primordia. The male-to-female flower ratio was 2.75/1, consistent with results obtained by other authors for this species (Freeman et al. 2007).

Flowering started in July in both years of our study. However, on Porquerolles Island close to Toulon (S France), plants originating from Algeria and Tunisia started flowering in September (Talamali et al. 2003), and in Morocco, Abbad and Benchaabane (2004) found that flowering commenced in mid-August in the Safi and Marrakech regions, and around the beginning of September in Ouarzazate. These two authors justified differences in flowering phenology by the variety of climatic conditions; high temperature, low humidity and periods without rainfall accelerate the formation of flower buds. Climate data show that the station in France is colder and rainier than the location we studied, with an annual mean temperature of 14.6°C with rainfall of 925.4 mm (Cuers Station: Rivas-Martínez and Rivas-Sáenz 1996–2009), but the locations under study in Morocco are hotter and drier, with an annual mean of 18.5°C and 317.0 mm in Safi, 19.9°C and 241.0 mm in Marrakech and 19.4°C and 101.4 mm in the Ouarzazate region (Rivas-Martínez and Rivas-Sáenz 1996–2009), so the climatic conditions cited do not explain the phenological delay observed in Morocco. This difference could be due to yearly variations in meteorological or microclimatic conditions.

The *Atriplex* species seems to be self-compatible (Heyligers 2001), and *A. halimus* is considered to be highly outbreeding (Haddioui and Baaziz 2001; Ferchichi et al. 2006), indicating that this species has effective mechanisms to prevent self-crossing. Protandry had been observed in this species by Abbad and Benchaabane (2004), who established a delay of nearly a month between the appearance of male and female flowers. In our study, we observed that male and female flowers seemed to open in the same weeks at population level (Figure 3), but the highest number of male flowers opened is reached one week before that of the female flowers, and that the upper, middle and lower parts of the inflorescence showed a similar pattern of behaviour (Figure 4). At plant and glomerule level (Figure 5), we see that male flowers opened one week before the females. This is in line with observations by Talamali et al. (2001), because female flowers are distributed along the distal parts of the dichasium and so open later than the males, which are mainly found on the proximal parts.

The start of fruit set and ovule development began when the stigmas were totally receptive. In 2006 the number of fruits per glomerule was significantly higher than in 2007, but seeds and bracts were much smaller in the first year. Abbad and Benchaabane (2004) observed that drought could cause a reduction in the number of female flowers in this species, so yearly variations in the number of fruits per glomerule could be explained by differences in rainfall between these years (Figure 1); the average accumulated rainfall from January to July in the location studied is 255.1 mm, while in 2006 and 2007 this value reached 327.0 and 175.6 mm respectively. This environmental effect has been observed in other species of *Atriplex* (McArthur 1977; McArthur and Freeman 1982).

The yearly variation in fruit size might be the result of physiological constraints including positional, temporal and environmental effects during offspring development; but most often offspring size is determined by an equal distribution of resources among the offspring (McGinley et al. 1987), so seed number could determine its weight (Smith and Fretwell 1974; Vaughton and Ramsey 1998; Sakai and Sakai 2005; Sadras 2007; Gambín and Borrás 2009; Kosiński 2010).

These size differences cannot be considered a case of seed heteromorphism, which occurs frequently in *Chenopodiaceae* (Imbert 2002), but it would be interesting to determine if these differences affect seed germination requirements, as occurs in species of *Atriplex* that show seed heteromorphism (Khan and Ungar 1984;1986), or if they influence individual plant performance, as in *Atriplex triangularis* Willd. (Ellison 1987. Kheiria *et al*. (2007) showed the existence of fruit polymorphism in Tunisian populations, the fruits from southern Tunisia being smaller than those collected from the central and northern areas. Fruit size recorded in our study for 2006 was similar to data collated by these authors in populations from central and northern Tunisia, but the size recorded in 2007 was bigger.

*A. halimus* fruits suffer high levels of pre-dispersal predation by insects. Larvae of *Coleophoridae* (*Lepidoptera*) were the most abundant predators of fruits and seeds in both years of the study. Huertas Dionisio (2007) found eight species of *Lepidoptera* on *A. halimus* plants, four of which are common in inflorescences: *Coleophora gaviaepennella* Toll, *C. granulatella* Zeller, *Goniodoma auroguttella* Zeller (fam. *Coleophoridae*) and *Gymnancyla sfakesella* Chrétien (fam. *Pyralidae*). This author also found pupals of the first and second species in the inflorescences in November, and *Gymnancyla sfakesella* larvae in September and October.

Huertas Dionisio (2005) describes the biological cycle of *Goniodoma auroguttella* in *A. halimus* plants in Huelva (SW Spain), including locations in the “Marismas del Odiel” Natural Park. Adults emerge in June, July and August and larvae feed on *Atriplex* fruits until October, gaining access to the ovary/achene by a hole made in the side of the valves, and moving along the inflorescence camouflaged by fruit valves. In October, the larvae go down the stem and bore a tunnel to the pith, where they transform into chrysalides. This cycle synchronizes with the previously described phenological phases (female flowering, fruit development and predation) observed in the present study. The anthesis of pistilate flowers occurs mainly in July and August, when adult insects are in flight; the first fruit predation event in both years was observed as the fruiting bracts reached maximum size, in the second half of September; peak predation is in October when larvae stop eating and the fruits begin to drop, which points to behavioural adaptation by the predator to exploit seed resources.

The yearly differences in levels of fruit predation could be explained by factors such as population size of the predators or variations in fruit and seed size. In 2006, seeds were smaller and the predation level was 62%, while in 2007 seeds were approximately 20% bigger and the predation level was 43% which suggests that when seeds are smaller, predators compensate by attacking more fruits. This is in line with the satiation mechanism proposed by Bonal et al. (2007), in which the satiation of small predators at seed level ensures that a proportion of the seeds survive predation.

In plants with sequential flowering, seed predation may be affected by the position of the fruit within the plant (Ehrlén 1996; Kudo et al. 2001; Sánchez et al. 2008). In *A. halimus* there were no significant differences in predation levels between different parts of the inflorescence. Mass fruiting appears to favour seed predators and allows them to attack the fruit on different parts of the inflorescence with the same frequency, but this strategy reduces fruiting time and ensures that some fruits avoid total seed destruction (Augspurger 1981; Pías and Guitián 2007; Gomes da Silva and Bezerra Pinheiro 2009). This strategy seems most appropriate when greater seed loss is caused by a specialist predator (Ims 1990), as is the case of the *Atriplex* population we have studied.

## Conclusions

At glomerule level, male flowers open earlier but protandry is incomplete because when the female flowers begin to be receptive, male flowers are still opening in the glomerule.

Fruit predation levels were 62.42 and 43.14% in 2006 and 2007 respectively. And larvae of Coleophoridae were the most abundant predators.

Different parts of the inflorescence showed a similar pattern of anthesis, and did not showed significant differences in predation levels.

## Authors’ original submitted files for images

Below are the links to the authors’ original submitted files for images. Authors’ original file for figure 1 Authors’ original file for figure 2 Authors’ original file for figure 3 Authors’ original file for figure 4 Authors’ original file for figure 5 Authors’ original file for figure 6 Authors’ original file for figure 7
