# Supplementary figures and images for: Reproductive phenology and pre-dispersal fruit predation in Atriplex halimus L. (Chenopodiaceae)
Source: Bot Stud. 2013 Aug 12;54:4. doi: 10.1186/1999-3110-54-4 (PMC5383916; doi:10.1186/1999-3110-54-4)

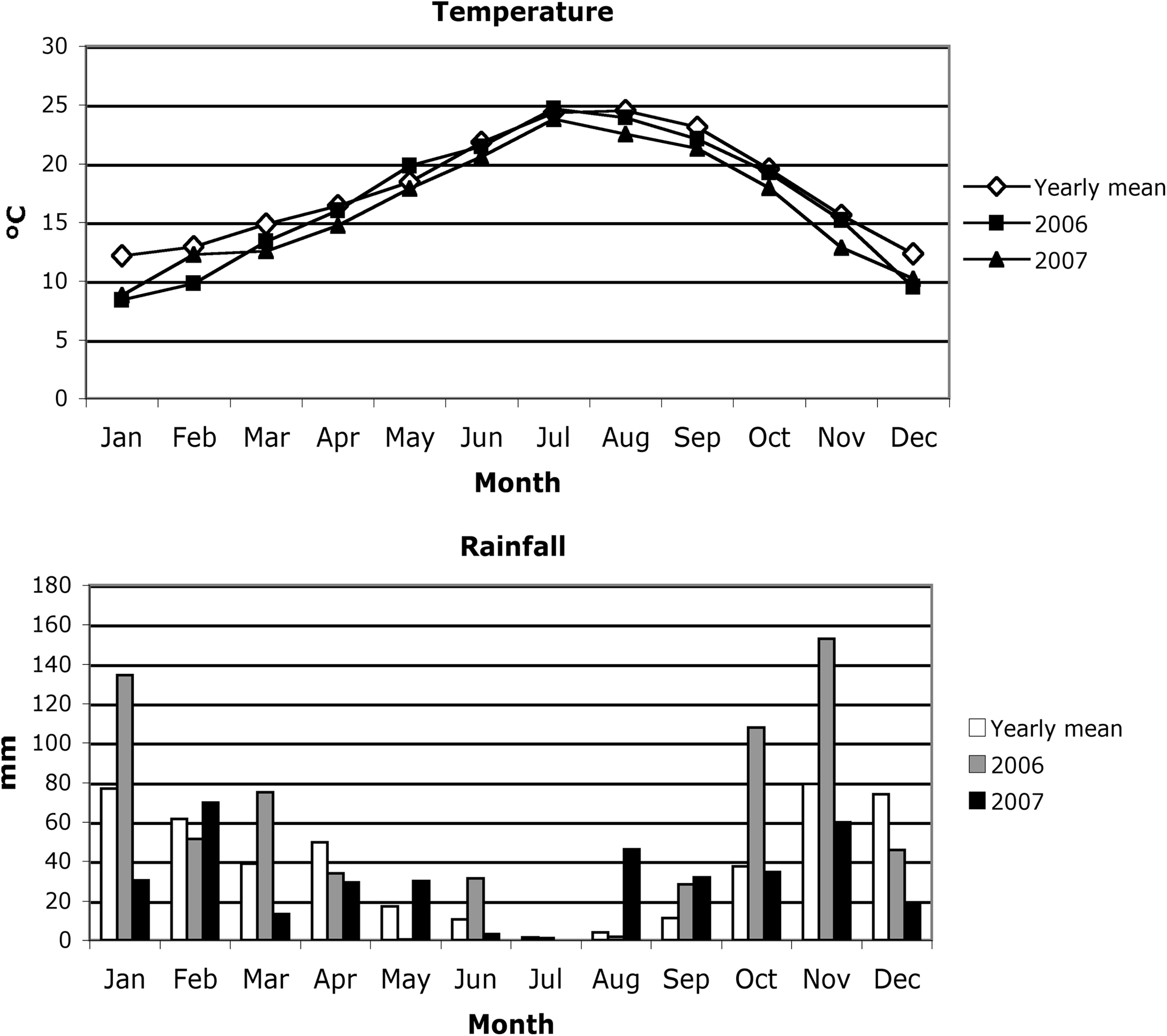

Supplement: Supplementary file 1 — Authors’ original file for figure 1 [file 40529_2012_4_MOESM1_ESM.tiff]

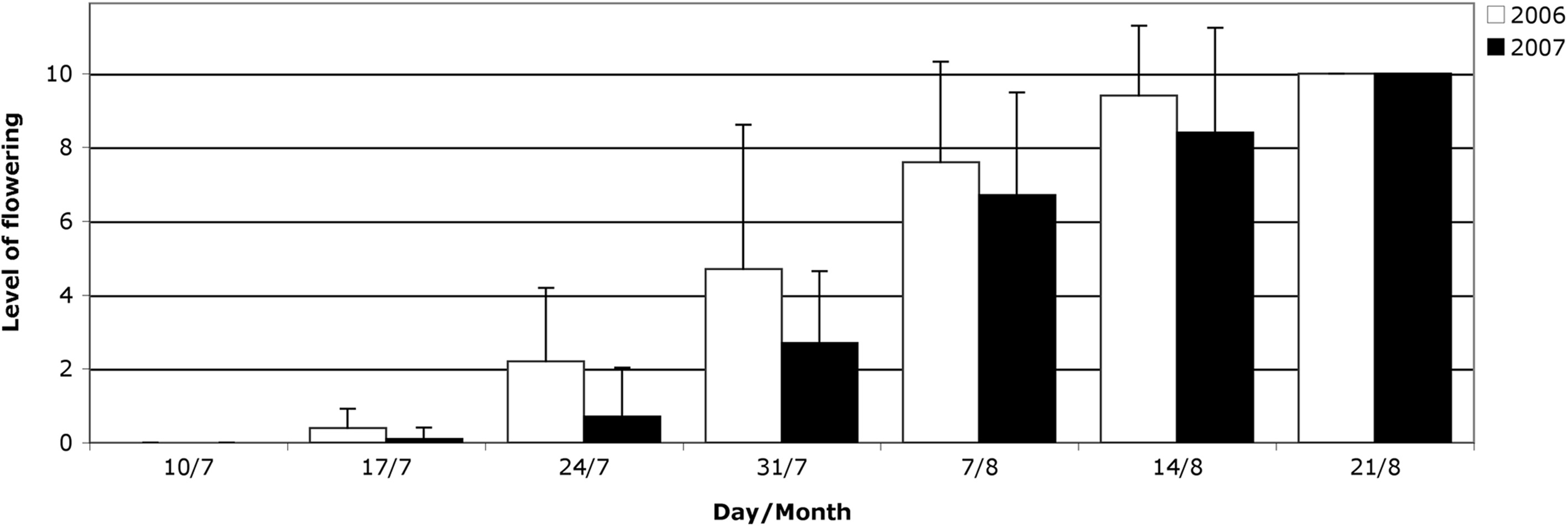

Supplement: Supplementary file 2 — Authors’ original file for figure 2 [file 40529_2012_4_MOESM2_ESM.tiff]

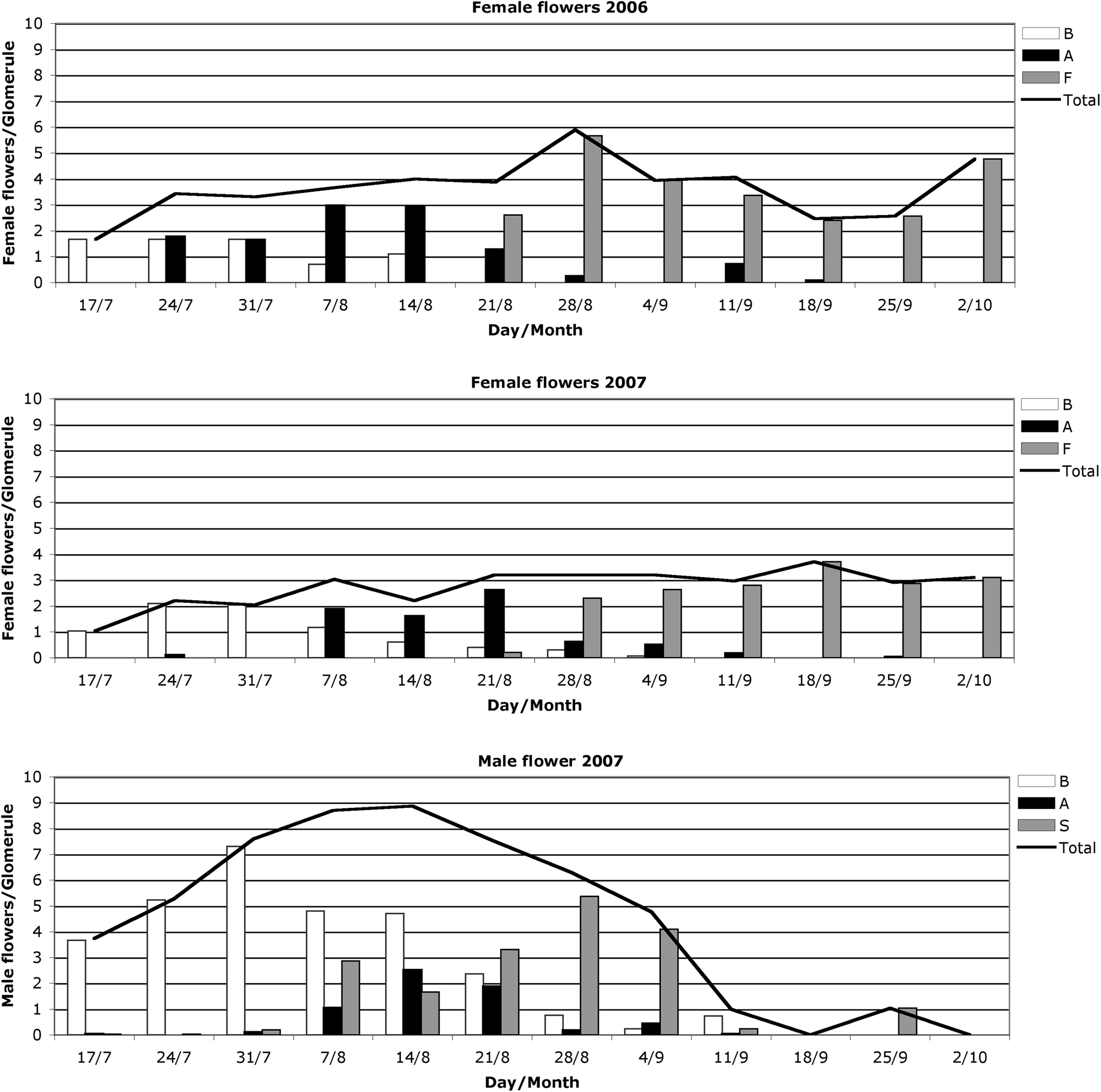

Supplement: Supplementary file 3 — Authors’ original file for figure 3 [file 40529_2012_4_MOESM3_ESM.tiff]

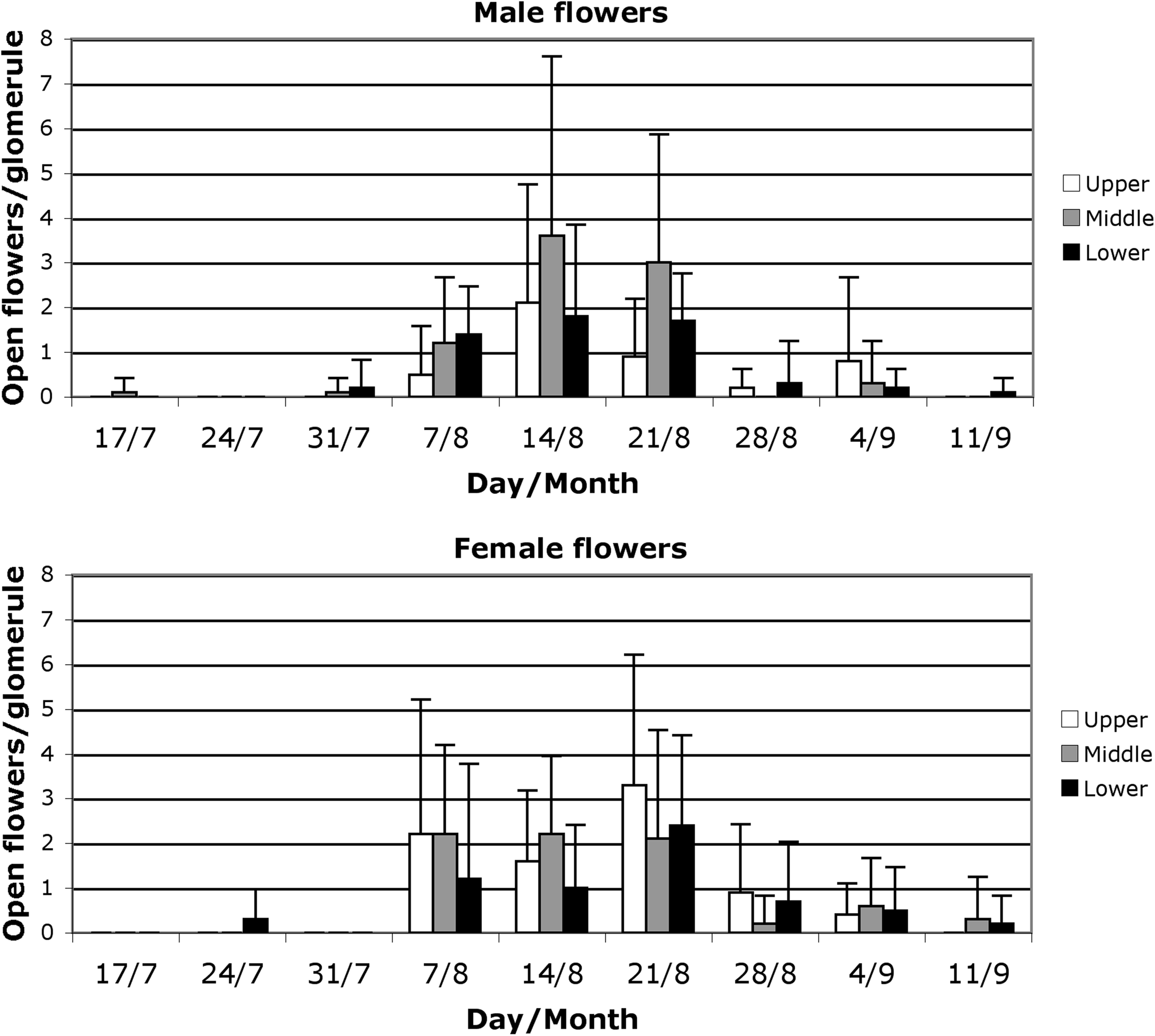

Supplement: Supplementary file 4 — Authors’ original file for figure 4 [file 40529_2012_4_MOESM4_ESM.tiff]

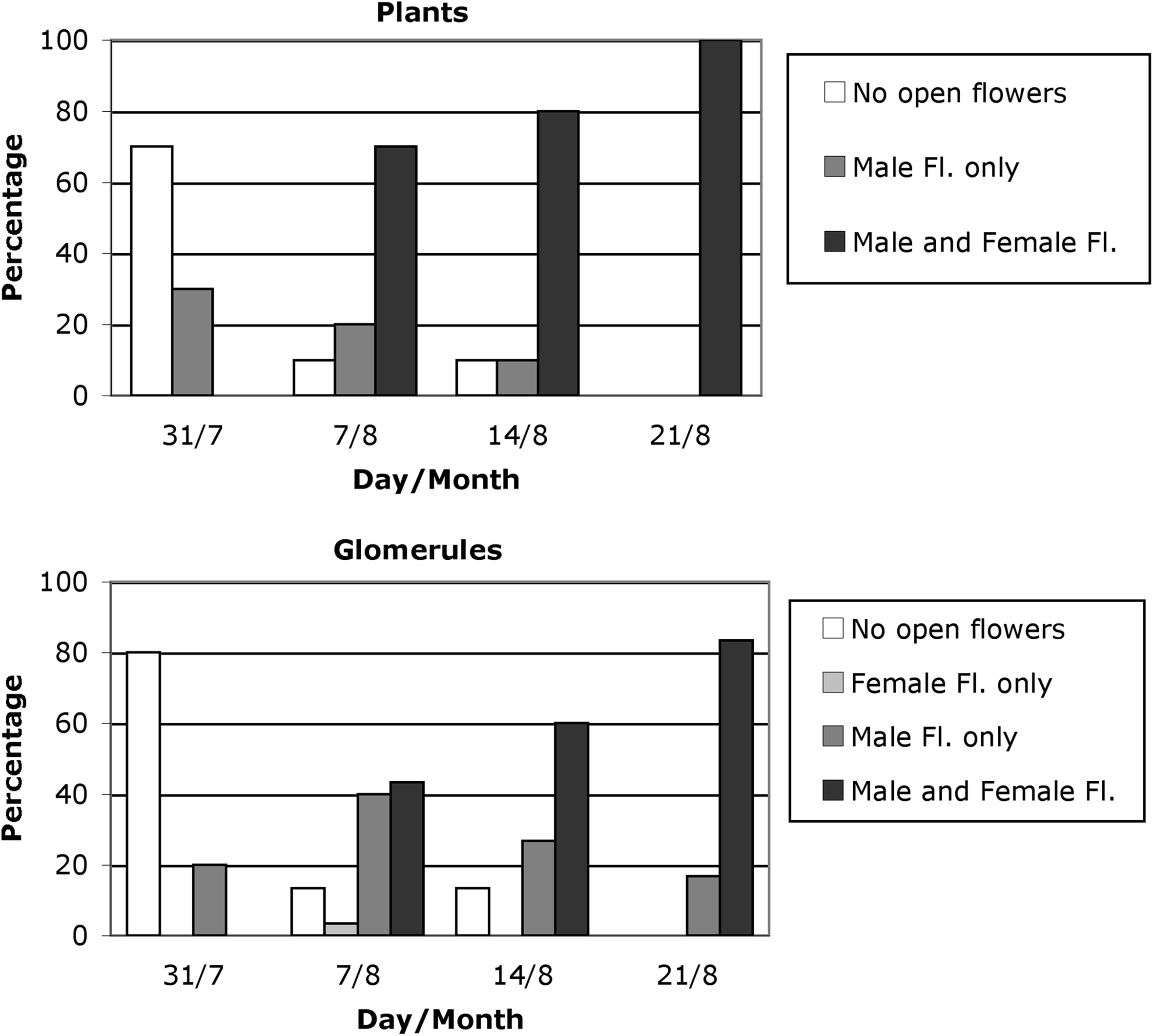

Supplement: Supplementary file 5 — Authors’ original file for figure 5 [file 40529_2012_4_MOESM5_ESM.tiff]

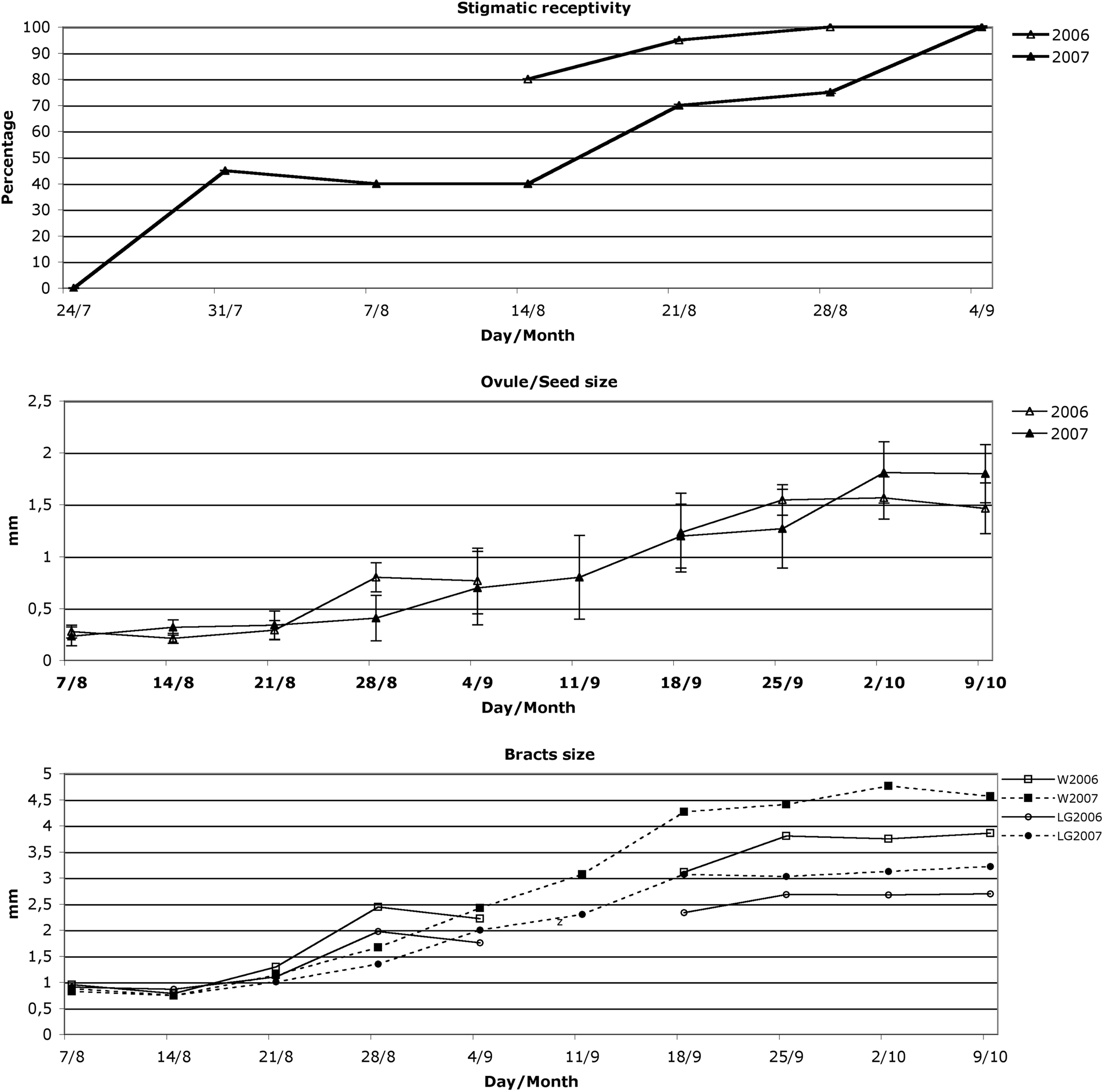

Supplement: Supplementary file 6 — Authors’ original file for figure 6 [file 40529_2012_4_MOESM6_ESM.tiff]

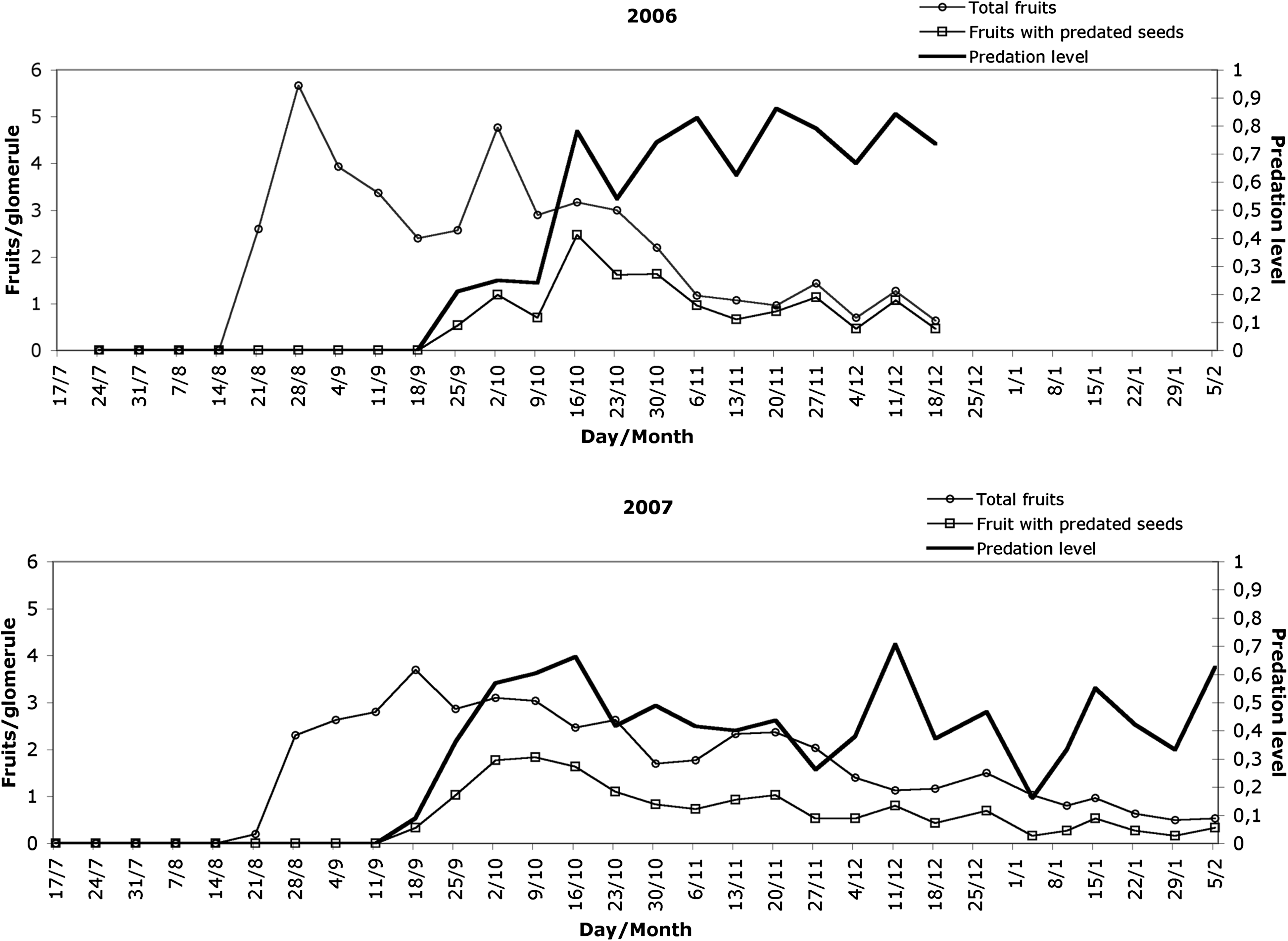

Supplement: Supplementary file 7 — Authors’ original file for figure 7 [file 40529_2012_4_MOESM7_ESM.tiff]
